# A Switch Role of Src in the Biphasic EGF Signaling of ER-Negative Breast Cancer Cells

**DOI:** 10.1371/journal.pone.0041613

**Published:** 2012-08-21

**Authors:** XinTian Zhang, Jun Meng, Zhao-Yi Wang

**Affiliations:** Department of Medical Microbiology and Immunology, Creighton University Medical School, Omaha, Nebraska, United States of America; II Università di Napoli, Italy

## Abstract

It is well established that epidermal growth factor (EGF) is a potent mitogen in cells expressing EGF receptor (EGFR). However, a body of evidence indicated that the effects of mitogenic EGF signaling exhibit a non-monotonic, or biphasic dose response curve; EGF at low concentrations elicits a mitogenic signaling pathway to stimulate cell proliferation while at high concentrations, EGF inhibits cell growth. However, the molecular mechanism underlying this paradoxical effect of EGF on cell proliferation remains largely unknown. Here, we investigated the molecular mechanisms underlying the biphasic EGF signaling in ER-negative breast cancer MDA-MB-231 and MDA-MB-436 cells, both of which express endogenous EGFR. We found that EGF at low concentrations induced the phosphorylation of the Src-Y416 residue, an event to activate Src, while at high concentrations allowed Src-Y527 phosphorylation that inactivates Src. EGF at 10 ng/ml also induced phosphorylation of the MAPK/ERK and activated cyclin D1 promoter activity through the Src/EGFR/STAT5 pathways but not at a higher concentration (500 ng/ml). Our results thus demonstrated that Src functions as a switch of EGF signaling depending on concentrations of EGF.

## Introduction

EGF is one of the most potent mitogens, which transmit signals for cell growth, survival and motility by binding to and activating the EGF receptor (EGFR) [1]–[3]. Amplification and mutation of the EGFR locus are frequently found in human epithelial malignancies 4–6. In breast cancer models, however, EGFR overexpression alone usually does not constitute efficient transformation and tumorigenesis while co-expression with the non-receptor kinase c-Src dramatically increases tumorigenesis 7–9. It has been reported that co-expression of EGFR and c-Src in breast cancer cell lines results in their association and c-Src-mediated phosphorylation of the EGFR at tyrosine 845 (Tyr845) within its catalytic domain, which contributes to enhanced cell proliferation and tumor formation *in vivo* [7]–[9]. EGFR-Tyr845 phosphorylation recruits the signal transducer and activator of transcription 3/5 (STAT3/5) that transmits the EGF signals to the cell nuclear and induces expression of the growth promoting genes such as c-Myc and cyclin D1 [10].

Like other growth stimulatory factors, the effects of mitogenic EGF signaling exhibit a non-monotonic, or biphasic dose response curve (inverted U-shaped); EGF at low concentrations, elicits a mitogenic signaling pathway to stimulate cell proliferation while at high concentrations, EGF inhibits cell growth and even induces cell apoptosis [11]. However, the molecular basis for this paradoxical effect of EGF on cell proliferation remains largely unknown. In some EGFR over-expressing cell lines such as vulva carcinoma A431 cells, EGF at pM range stimulates cell proliferation while EGF at nM range induces growth inhibition, terminal differentiation and apoptosis [12]–[14]. Previously, it was shown that EGF-induced growth arrest and apoptosis is associated with the activation of STAT1, which in turn activates p21/WAF1 and Caspase 1 [15]–[19]. In addition, the tyrosine kinase Etk/Bmx, a downstream signaling molecule of EGF pathway is involved in the EGF-induced activation of STAT1 and apoptosis [20].

In the current study, we investigated the molecular mechanisms underlying the biphasic or non-monotonic EGF signaling in ER-negative breast cancer cells that express endogenous EGFR and revealed the involvement of the Src/EGFR/STAT5 signaling pathway in the biphasic EGF signaling.

## Materials and Methods

### Chemicals and Antibodies

EGF was purchased from Sigma Chemical Co. (St. Louis, MO). The Src inhibitor dasatinib was obtained from LC Laboratories (Woburn, MA). The Src inhibitor PP2, the EGFR inhibitor AG1478 and the PI3K inhibitor LY294002 were from Tocris Bioscience (Ellisville, MO). Phospho-EGFR and -Src antibodies, EGFR and Src antibodies, anti-phospho-p44/42 ERK (Thr202/Tyr204) (197G2) mouse monoclonal antibody (mAb) and anti-p44/42 ERK (137F5) rabbit mAb were purchased from Cell Signaling Technology (Boston, MA). Antibody of cyclin D1 was purchased from Santa Cruz Biotechnology (Santa Cruz, CA).

### Cell Culture, Treatment and Growth Assay

MDA-MB-231 and MDA-MB-436 cells were obtained from American Type Culture Collection (ATCC, Manassas, VA). Cells were maintained at 37°C in a 10% CO_2_ atmosphere in DMEM and 10% fetal calf serum in a humidified incubator. For ERK activation assays, cells were treated with vehicle PBS or indicated concentrations of EGF. To test the effects of different inhibitors, all inhibitors were added 10 min. before EGF addition.

To examine cell growth in the presence or absence of different concentrations of EGF, cells maintained in serum-free medium overnight were treated with different concentrations of EGF or PBS vehicle as a control. The cells were seeded at 1×10^4^ cells per dish in 60 mm dishes and the cell numbers were determined using the ADAM automatic cell counter (Digital Bio., Korea) after seven days. Five dishes were used for each treatment and experiments were repeated more than three times.

### Plasmids, DNA Transfection and Luciferase Assay

The expression vectors for a dominant-negative mutant of Src (pCMV5/SrcK295) and a constitutively active mutant of Src (pCMV5/SrcY527F) were obtained from Dr. Yun Qiu at the Department of Pharmacology and Experimental Therapeutics, University of Maryland School of Medicine. Dr. Linda Schuler at Department of Comparative Biosciences, University of Wisconsin-Madison, kindly provided the luciferase reporter plasmids of the cyclin D1 promoter carrying GAS1 and 2 mutations. Two naturally occurring dominant-negative STAT5 mutants, Stat5aΔ713 and Stat5aΔ740 were provided by Dr. Hiroko Yamashita at Department of Surgery II, Nagoya City University. The wild-type luciferase reporter plasmid of the cyclin D1 promoter, cyclin D1 pl-963 was obtained from Dr. Chris Albanese at Departments of Oncology and Pathology, Georgetown University Medical Center. LHRR, a consensus STAT5 reporter construct containing a six-repeat sequence of the lactogenic hormone response element (LHRE) was kindly provided by Dr. Sarah Parson at Department of Microbiology, University of Virginia Health System. Cells were all co-transfected with a cytomegalovirus-driven *Renilla* luciferase plasmid, pRL-CMV (Promega, Madison, WI) to establish transfection efficiency. Twenty-four hours after transfection, cells were treated withorwhiout the indicated inhibitors for twenty-four hours. Forty-eight hours after transfection, cell extracts were prepared and luciferase activities were determined and normalized using the Dual-Luciferase Assay System (Promega, Madison, WI) and a TD 20/20 Luminometer (Turner BioSystems, Inc., Sunnyvale, CA) as instructed by the manufacturer.

### Western Blot Analysis

For Western blot analysis, cells washed with ice-cold PBS were lysed with the lysis buffer (50 mM Tris-HCl pH 8.0, 150 mM NaCl, 0.25 mM EDTA pH 8.0, 0.1% SDS, 1% Triton X-100, 50 mM NaF) supplemented with the protease and phosphatase inhibitors (Sigma, St. Louis, MO). The protein amounts were measured using the DC protein assay kit (BIO-RAD Laboratories, Hercules, CA). The same amounts of the cell lysates were boiled for 5 minutes in loading buffer and separated on a SDS-PAGE gel. After electrophoresis, the proteins were transferred to a PVDF membrane. The membranes were probed with various primary antibodies, HRP-conjugated secondary antibodies, and visualized with enhanced chemiluminescence (ECL) detection reagents (GE Healthcare Bio-Sciences Corp., Piscataway, NJ).

### Immunoprecipitation and Immunoblot Analysis

For imunoprecipitation assays, cells were washed twice with ice-cold PBS and lysed with the lysis buffer (150 mM NaCl, 20 mM TrisHCl, pH 7.4, 0.1% NP-40) supplemented with the protease and phosphatase inhibitors (Sigma). Cell lysates were then incubated with the indicated antibodies, or pre-immune serum and immunoprecipitated with protein A/G plus agarose. The precipitates were then washed extensively, separated on SDS-PAGE and analyzed with Western blot analysis.

### Statistical Analysis

Data were summarized as the mean ± standard error (SE) using the GraphPad InStat software program (GraphPad Software, La Jolla, CA, USA). Tukey-Kramer Multiple Comparisons Test was also used, and the significance was accepted for *P*<0.05.

## Results

### ER-negative breast cancer cells exhibit biphasic EGF signaling

**Figure 1:**
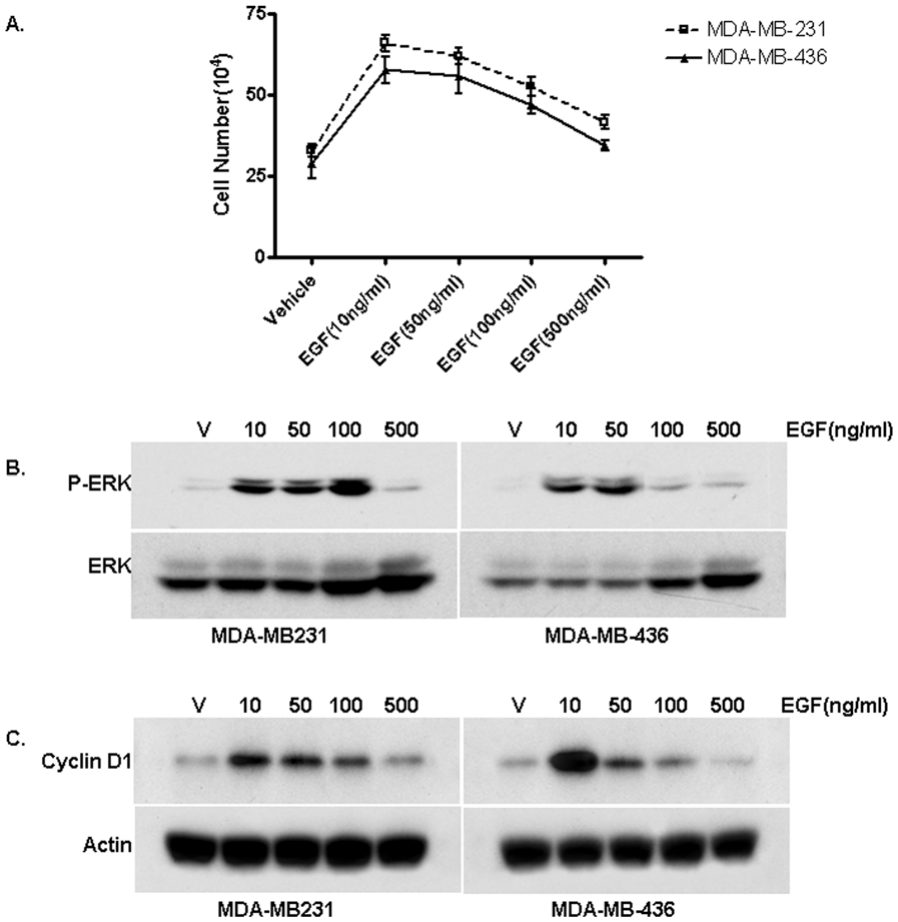
ER-negative Breast Cancer Cells Exhibit Biphasic EGF Signaling. (A). The effects of EGF on the proliferation rate of MDA-MB-231 and MDA-MB-436 cells. Serum starved cells were treated with indicated concentrations of EGF or PBS as a control. The cell numbers were determined using an automatic cell counter after seven days. Five dishes were used for each concentration and experiments were repeated more than three times. The mean cell number ± SE are shown. (B). The dose-dependent pattern of EGF-stimulated phosphorylation of the MAPK/ERK1/2 in MDA-MB-231 and MDA-MB-436 cells. Starved cells were treated with indicated doses of EGF for 15 min. Western blot analysis was performed to assess induction of ERK1/2 phosphorylation. The experiment was repeated more than three times. The representative results are shown. (C). The dose dependent induction cyclin D1 by EGF in MDA-MB-231 and MDA-MB-436 cells. The experiment was repeated more than three times. The representative results are shown.

To probe the underlying mechanisms of the biphasic EGF signaling, we decided to first determine the growth rate of two ER-negative breast cancer cell lines MDA-MB-231 and MDA-MB-436 in response to different concentrations of EGF. As shown in Figure 1A, the ER-negative breast cancer cells treated with 10 ng/ml EGF exhibited an increased growth rate compared with cells treated with PBS. The dose-response curves of EGF exhibited a biphasic pattern; increasing concentrations that initially stimulated cell growth but failed to do so at higher concentrations (Figure 1A). Our data thus indicated that EGF-induced cell growth exhibited a non-monotonic or biphasic dose-response curve in these ER-negative breast cancer cells.

### EGF induces biphasic activation of the MAPK/ERK and cyclin D1 expression in ER-negative breast cancer cells

We then examined EGF-induced phosphorylation of the MAPK/ERK1/2 in these two cell lines. We treated cells with EGF at different concentrations for 15 min. Western blot analysis with a phospho-specific ERK1/2 antibody was performed to assess the phosphorylation levels of the ERK1/2. As shown in Figure 1B, we found that EGF was able to induce the activation of the MAPK/ERK at 10 ng/ml while failed to activate the MAPK/ERK at 500 ng/ml in both ER-negative breast cancer cell lines, consistent with the biphasic growth pattern of the dose-response curves of these cells to EGF. Additionally, we found a biphasic induction pattern of cyclin D1 expression in the cells treated with different concentrations of EGF (Figure 1C).

### The Src/EGFR/STAT5 are involved in biphasic EGF signaling

**Figure 2:**
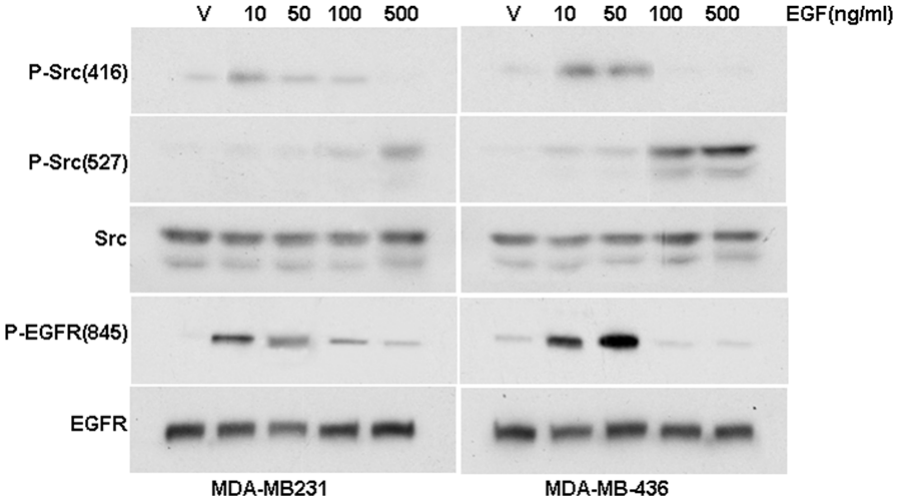
Different concentrations of EGF induce Src phosphorylation at distinct residues. Western blot analysis of the effects of different concentrations of EGF on the phosphorylation levels of EGFR-Y845, Src-Y416 and Src-Y527 in MDA-MB-231 and MDA-MB-436 cells.

It is well established that EGF treatment activates Src, which then phosphorylates EGFR-Tyr-845 [21], [22]. We then examined the phosphorylation status of Src-Tyr-416 and EGFR-Tyr-845 in these cells treated with different concentrations of EGF. Figure 2 shows that in MDA-MB-231 and MDA-MB-436 cells, 10 ng/ml EGF elicited phosphorylation of Src-Tyr-416 and EGFR-Tyr-845 while failed to do so with increased concentrations. Intriguingly, phosphorylation of Src-Tyr-527, an event associated with inactivation of Src activity, was not observed in the cells treated with 10 ng/ml EGF but was obvious in cells treated with 500 ng/ml EGF (Figure 2). These results suggested that EGF at low concentrations induces phosphorylation of Src-Y-416 and activated Src whereas at high concentrations of EGF, Src-Y-527 is phosphorylated that inactivates Src activity.

**Figure 3:**
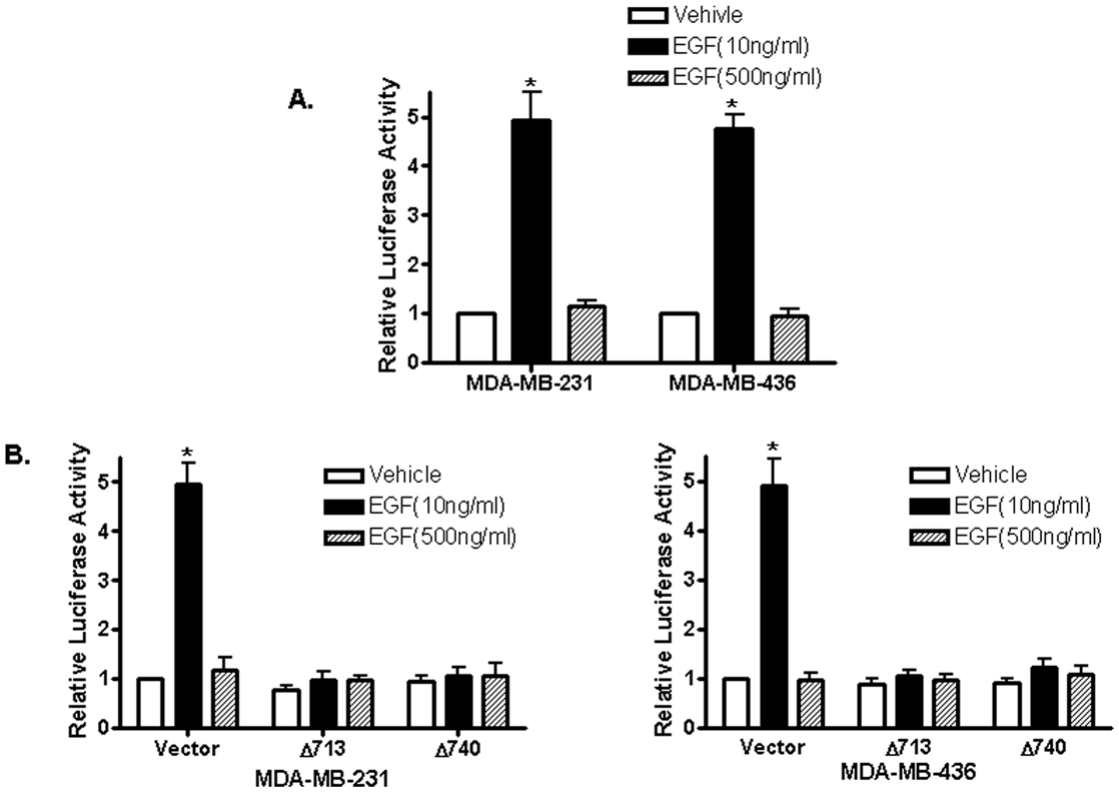
EGF induces biphasic STAT5 activities in ER-negative breast cancer cells. (A). ER-negative breast cancer cells were transfected with the luciferase reported plasmid LHRR-Luc that containing six copies of STAT5-binding sites. Transfected cells were treated with PBS, 10 ng/ml or 500 ng/ml of EGF. The luciferase activities were assayed and normalized using a cytomegalovirus-driven *Renilla* luciferase plasmid. Columns: means of the relative luciferase activity from four independent experiments. Luciferase activity in transfected cells treated with vehicle is arbitrarily set as 1.0; bars, SE. *, *P*<0.05, for cells treated with PBS vehicle vs 10 ng/ml of EGF. (B). Cells were transfected with the LHRR-Luc reporter together with an empty expression vector (vector) and the expression vectors of two dominant-negative STAT5a mutants carrying truncations at their C-terminal (STAT5aΔ713 and STAT5aΔ740) before treated with PBS vehicle (V), 10 ng/ml or 500 ng/ml µM of EGF. Columns: means of the relative luciferase activity from three independent experiments. Luciferase activity of cells co-transfected with an empty expression vector and treated with vehicle is arbitrarily set as 1.0; bars, SE. *, *P*<0.05, for cells treated with PBS vehicle vs 10 ng/ml of EGF.

It was reported that signal transducer and activator of transcription 5 (STAT5), c-Src and EGFR are involved in EGF-stimulated cell proliferation [10]. To examine whether STAT5 is involved in the observed biphasic EGF signaling, we transfected MDA-MB-231 and MDA-MB-436 cells with a consensus STAT5 reporter construct, containing a six-repeat sequence of the lactogenic hormone response element (LHRE) [23] and treated with EGF at 10 ng/ml and 500 ng/ml We found that 10 ng/ml EGF potently activates the promoter activity of the reporter plasmid while 500 ng/ml EGF failed to do so (Figure 3A), suggesting that EGF at low concentrations was able to activate STAT5 protein-mediated transcription. To confirm the involvement of STAT5, we also included two naturally occurring dominant-negative mutants of STAT5 (Stat5aΔ713 and Stat5aΔ740) described before [24] in the transient transfection assays, and found that both dominant-negative mutants of STAT5 potently attenuated 10 ng/ml EGF-induced promoter activity, indicating that STAT5 is involved in the biphasic EGF signaling of these ER-negative breast cancer cells (Figure 3B).

### Src is involved in biphasic cyclin D1 expression in response to different concentrations of EGF

**Figure 4:**
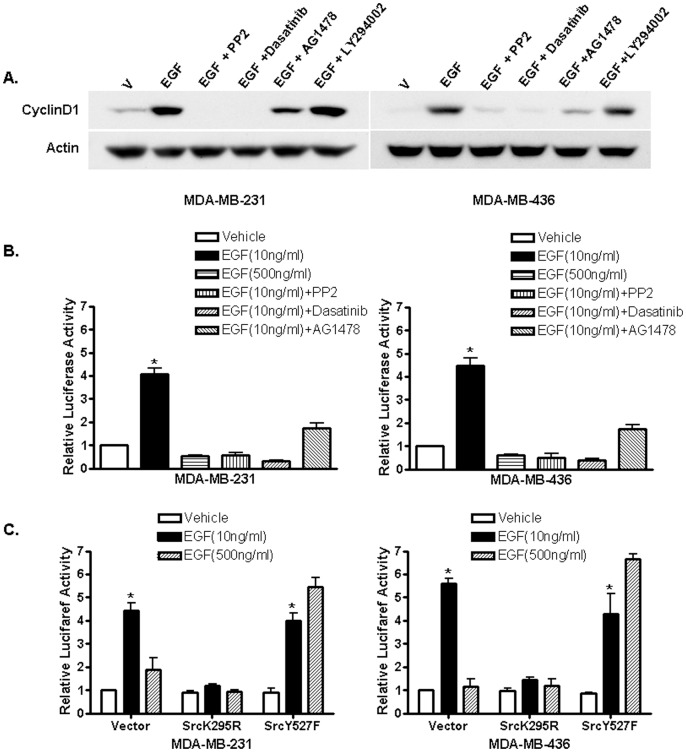
Src is involved in EGF induction of cyclin D1. (A). Western blot analysis of cyclin D1 expression in MDA-MB-231 and -436 cells. Cells were treated with vehicle (PBS) and EGF alone or together with the Src inhibitors PP2 and Dasatinib, the EGFR inhibitor AG1478 and PI3K inhibitor LY294002. Cell lysates were analyzed with anti-cyclin D1 antibody and anti-actin antibody was used to ensure equal loading. The experiment was repeated three times, and the representative results are shown. (B). Src inhibitors inhibit EGF induction of cyclin D1 promoter activity. ER-negative breast cancer cells were transfected with the luciferase reported plasmid cyclin D1 pl-963 that containing a luciferase gene driven by the cyclin D1 promoter. Transfected cells were treated with vehicle (PBS), 10 ng/ml or 500 ng/ml of EGF and 10 ng/ml EGF plus different inhibitors. The luciferase activities were assayed and normalized using a cytomegalovirus-driven Renilla luciferase plasmid. Columns: means of the relative luciferase activity in cells treated with vehicle that is arbitrarily set as 1.0 from four independent experiments; bars, SE. *, *P*<0.05, for cells treated with vehicle (PBS) vs 10 ng/ml of EGF. (C). The involvement of Src in EGF induction of cyclin D1 promoter activity. Cells were transfected with the luciferase reported plasmid cyclin D1 pl-963 together with an empty expression vector or Src mutants, a dominant-negative mutant (SrcK295R) and a constitutively active mutant (SrcY527F). Transfected cells were treated with vehicle (PBS), 10 ng/ml or 500 ng/ml of EGF. The luciferase activities were assayed and normalized using a cytomegalovirus-driven *Renilla* luciferase plasmid. Columns: means of the relative luciferase activity from four independent experiments. Luciferase activity in transfected cells treated with vehicle is arbitrarily set as 1.0; bars, SE. *, *P*<0.05, for cells treated with vehicle (PBS) vs 10 ng/ml of EGF.

In the experiments described above, we observed that the cells treated with different concentrations of EGF also exhibited biphasic patterns of cyclin D1 induction. We then decided to determine whether the Src signaling pathway is involved in the biphasic cyclin D1 induction by EGF. We first tested if the Src inhibitors PP2 and Dasatinib were able to inhibit cyclin D1 induction by 10 ng/ml EGF. Cells were treated with EGF and together with PP2, Dasatinib, the EGFR inhibitor AG1478 and the PI3K inhibitor LY294002, and Western blot analysis was performed to examine cyclin D1 expression. Figure 4A shows that EGF-induced cyclin D1 expression was strongly blocked by both Src inhibitors, weakly with AG1478 while Ly294002 was without effect, suggesting that Src is involved in EGF induction of cyclin D1 expression (Figure 4A).

To examine whether induction of the cyclin D1 promoter activity by EGF also exhibited a biphasic pattern, we first transfected both cell lines with a human cyclin D1 promoter-luciferase construct and then treated transfected cells with 10 ng/ml or 500 ng/ml EGF. We found that 10 ng/ml EGF was able to induce cyclin D1 promoter activity whereas 500 ng/ml EGF failed to induce cyclin D1 promoter activity (Figure 4B), indicating that the biphasic effects of EGF on induction of cyclin D1 expression is through regulation of its promoter activity. We also found both Src inhibitors potently inhibited 10 ng/ml of EGF-induced promoter activity and the EGFR inhibitor AG1478 had less effect, suggesting Src is involved in EGF-induced cyclin D1 promoter activity.

To further confirm the involvement of Src in EGF-regulated cyclin D1 expression, these ER-negative breast cancer cells were co-transfected with the cyclin D1 promoter reporter plasmid and pCMV5/SrcK295M, a dominant-negative mutant of Src or pCMV5/SrcY527F, a constitutively active mutant of Src, respectively. We found that co-transfection of the dominant-negative mutant of Src abrogated the cyclin D1 promoter activity induced by 10 ng/ml EGF while had no effects in cells treated with 500 ng/ml EGF (Figure 4C). On the contrary, the constitutively active mutant of Src restored the cyclin D1 promoter activity suppressed by 500 ng/ml of EGF (Figure 4C). These results indicated Src plays an integral role in biphasic response of cyclin D1 expression to different concentrations of EGF.

### STAT5 is involved in EGF-induced cyclin D1 promoter activity

**Figure 5:**
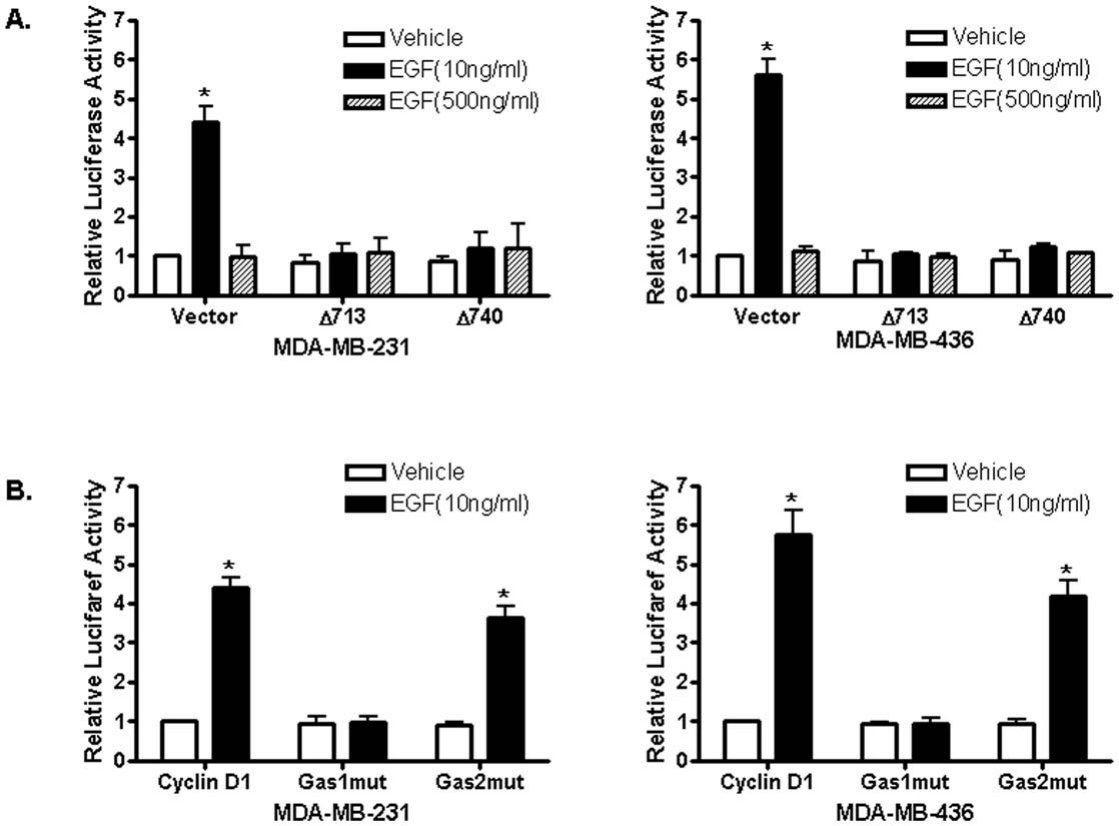
STAT5 is involved in EGF induction of cyclin D1 promoter activity. (A). The involvement of STAT5 in EGF induction of cyclin D1 promoter activity. Cells were transfected with the luciferase reported plasmid cyclin D1 pl-963 together with an empty expression vector or two dominant-negative STAT5a mutants, STAT5aΔ713 and STAT5aΔ740. Transfected cells were treated with vehicle (PBS), 10 ng/ml or 500 ng/ml of EGF. Columns: means of the relative luciferase activity from four independent experiments. Luciferase activity in transfected cells transfected with an empty expression vector and treated with vehicle is arbitrarily set as 1.0; bars, SE. *, *P*<0.05, for cells treated with vehicle (PBS) vs 10 ng/ml of EGF. (B). The GAS1 site is involved in induction of the cyclin D1 promoter activity by EGF. Cells were transiently transfected with either the wild-type cyclin D1 promoter or the same promoter construct containing mutated GAS1 (GAS1mut) or GAS2 (GAS2mut) sequence, respectively. Transfected cells were treated with PBS vehicle or 10 ng/ml of EGF, and the luciferase activity was presented relative to the wild-type cyclin D1 promoter-transfected cells treated with PBS that is arbitrarily set as 1.0. *, *P*<0.05, for cells treated with vehicle (PBS) vs 10 ng/ml of EGF.

Previously, it was reported that the STAT proteins are involved in regulation of the cyclin D1 promoter activity through their interaction with the two consensus gamma-interferon-activation sites (GAS) sites located in the cyclin D1 promoter [25]. We decided to examine whether STAT5 is involved in cyclin D1 promoter activity induced by 10 ng/ml EGF in these ER-negative breast cancer cells. Two dominant-negative mutants of STAT5a were co-transfected with the cyclin D1 promoter reporter plasmid, and transfected cells were treated with 10 ng/ml EGF. We found that inclusion of both STAT5a mutants strongly suppressed the cyclin D1 promoter activity induced by 10 ng/ml EGF (Figure 5A), suggesting that low concentrations of EGF induced the cyclin D1 promoter activity through the Src/EGFR/STAT5 pathway in these ER-negative breast cancer cells.

The human cyclin D1 promoter harbors binding sites for a number of transcription factors. There are two gamma-interferon-activation sites (GAS) located at −457 and −224 (relative to the transcription initiation site) of the cyclin D1 promoter region that can be recognized by STAT5 protein [25]. To assess the involvement of the two GAS sequences in EGF-induced cyclin D1 promoter activity, we transfected these ER-negative breast cancer cells with two mutants of the cyclin D1 promoter/luciferase constructs, GAS1mut and GAS2mut that mutated the two GAS sequences located at −457 and −224, respectively to prevent STAT protein binding (25). The promoters containing GAS1 mutation failed to respond to 10 ng/ml EGF while GAS2 mutation had no significant effect (Figure 5B), indicating that the GAS1 site is involved in EGF-induced cyclin D1 promoter activity.

### Different concentrations of EGF affect the association of EGFR and Src differently

**Figure 6:**
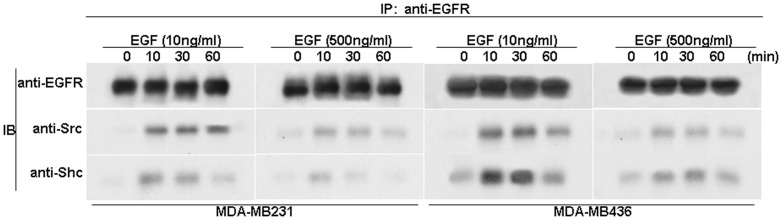
Different concentrations of EGF affect the association of EGFR and Src differently. Co-immunoprecipitation and Western blot analysis of EGFR and Src in MDA-MB-231 cells. Cells were treated with different concentrations of EGF for different time periods were lysed and the cell lysates were immunoprecipitated with pre-immune and indicated antibodies. The immunoprecipitates were blotted by indicated antibodies.

To elucidate the molecular mechanism by which different concentrations of EGF influence Src phosphorylation, we examined the effects of different concentrations of EGF on the association of EGFR with Src and Shc. Both cell lines were treated with 10 or 500 ng/ml of EGF for different time periods. Cell lysates were immunoprecipitated with pre-immune or anti-EGFR, and blotted by anti-EGFR, anti-Src or anti-Shc antibodies. Figure 6 shows that at l0 ng/ml, EGF strongly induced association of EGFR with Shc and Src, which was significantly decreased when treated with 500 ng/ml of EGF (Figure 6). Pre-immune antibody failed to immunoprecipitate any of these proteins (data not shown). Our results thus demonstrated that EGF at different concentrations regulates the association of EGFR with Shc and Src differently.

## Discussion

It is well recognized that EGF is one of the most potent growth factors that transmits signals for cell growth, survival and sometimes motility by engaging the EGFR [1]–[3]. However, in squamous carcinoma A431 cells, which express high levels of endogenous EGFR, EGF at pM range stimulates cell growth, while at nM range inhibits proliferation, arrests cell cycle, alters cdk2 activity by induction of p21, and even induces cell apoptosis [14]–[19]. Here, we found that EGFR at higher concentrations failed to stimulate cell proliferation in ER-negative breast cancer cells. We also observed that at high concentrations, EGF failed to activate the MAPK/ERK pathway and to induce the growth-promoting gene cyclin D1. Thus, activation of the MAPK/ERK signaling and cyclin D1 expression mediated by EGF signaling also exhibited a biphasic pattern, which provided a molecular explanation to the observed biphasic EGF signaling.

Unlike the EGFR-over-expressing cells such as A431 cells, we did not observe strong cell apoptosis under the highest concentrations of EGF, 500 ng/ml, we used. We also used higher concentrations of EGF to observe growth inhibition in these ER-negative breast cancer cells compared to the concentrations used in A431 cells [14]–[19]. We speculate that the difference of the levels of EGFR expression between these cell lines may provide an explanation to this discrepancy; higher levels of receptor expression may require lower concentrations of EGF to confer growth inhibition, i.e. the concentration dependent growth curve may shift to the left.

To probe the underlying mechanisms of the biphasic estrogen signaling, we found that at 10 ng/ml, EGF induced phosphorylation of Src at Tyr-416. Intriguingly, phosphorylation of Src-Tyr-527 was observed in cells treated with EGF at 500 ng/ml. The Src protein has three major domains, SH2 (for Src homology 2), SH3, and the kinase catalytic domain. Both SH2 and SH3 domains play a role in protein-protein interactions, while the kinase catalytic domain contains the kinase active site. Src can be switched from an inactive to an active state through control of its phosphorylation state. Src-Tyr-416 can be auto-phosphorylated, which activates Src by displacing the P-Tyr-416 from the binding pocket, allowing the substrate to gain access. However, phosphorylation of Tyr-527 inactivates Src through the interaction of P-Tyr-527 with the SH2 domain, which effectively folds Src up into a closed, inactive state. Our results thus demonstrated, for the first time, that phosphorylation state of Src-Y-416 and-Y-527 acts as a switch to turn on and off the EGF signaling depending on concentrations of EGF.

At present, not much is known about how different concentrations of EGF induce phosphorylation of either Tyr-416 or Tyr-527. Previously, it has been reported that EGF stimulation leads to interaction of EGFR and Src as well as Src-Tyr-416 auto-phosphorylation [26]. The Src-Tyr-527 is a critical site for regulation of Src activity, which can be phosphorylated and dephosphorylated by various proteins [27], such as CSK kinase (phosphorylates), or SHP-1 phosphorylase (dephosphorylates). Thus, the phosphorylation state of Src-Tyr-527 is dynamically and strictly regulated by phosphorylation and dephosphorylation. We found that at 10 ng/ml, EGF triggered a strong interaction between EGFR and Shc/Src while at 500 ng/ml, EGF failed to induce a strong interaction between EGFR and Shc/Src. At present, it can only be speculated that different concentrations of EGFR may trigger different conformations of EGFR, which will then alter the interaction between EGFR and Shc/Src and activate either kinase or phosphatase to regulate phosphorylation of Src-Tyr-527.

We also showed that at 10 ng/ml, EGF induced Src-mediated phosphorylation of the EGFR-Tyr-845 residue and STAT5-mediated activation of the cyclin D1 promoter activity while at 500 ng/ml, EGF failed to do so, which provided a molecular explanation for the loss of the growth promoting activity observed in cells treated with high concentrations of EGF. Previously, it was reported that in EGF treated A431 cells, EGF-induced Src activation and Src-dependent phosphorylation of EGFR-Tyr-845 recruits STAT proteins as downstream effectors of phosphorylated EGFR-Tyr-845 [19], . Consistent with these findings, our observations demonstrated that the EGFR/Src/STAT5 pathway is involved in the biphasic EGF signaling in ER-negative breast cancer cells.

In summary, we have shown that ER-negative breast cancer cells exhibited biphasic EGF signaling and Src acts as a switch to turn on/off the mitogenic EGF signaling depending on concentrations of EGF. In our experiments, we also observed that EGF at higher concentrations strongly induced the levels of p21^waf1^ expression (data not shown), suggesting that the induction of p21^waf1^ is also involved in growth inhibition observed in ER-negative breast cancer cells treated with high concentrations of EGF. Thus, further study will be directed to dissect the molecular mechanisms underlying the induction of p21^waf1^ expression by different concentrations of EGF and its physiological consequences in these ER-negative breast cancer cells.
